# CD4 and FOXP3 as predictive markers for the recurrence of T3/T4a stage II colorectal cancer: applying a novel discrete Bayes decision rule

**DOI:** 10.1186/s12885-022-10181-7

**Published:** 2022-10-18

**Authors:** Yuki Nakagami, Shoichi Hazama, Nobuaki Suzuki, Shin Yoshida, Shinobu Tomochika, Hiroto Matsui, Yoshitaro Shindo, Yukio Tokumitsu, Satoshi Matsukuma, Yusaku Watanabe, Michihisa Iida, Ryouichi Tsunedomi, Shigeru Takeda, Tomonobu Fujita, Yutaka Kawakami, Hiroyuki Ogihara, Yoshihiko Hamamoto, Tatsuya Ioka, Tsuyoshi Tanabe, Tomio Ueno, Hiroaki Nagano

**Affiliations:** 1 Department of Translational Research and Developmental Therapeutics Against Cancer, Yamaguchi University School of Medicine, Ube, Yamaguchi, Japan; 2 Department of Gastroenterological, Breast and Endocrine Surgery, Yamaguchi University Graduate School of Medicine, 1-1-1 Minami-Kogushi, Ube, Yamaguchi, 755-8505 Japan; 3 Department of Public Health and Preventive Medicine, Yamaguchi University Graduate School of Medicine, Ube, Yamaguchi, Japan; 4 Division of Cellular Signaling, Institute for Advanced Medical Research, Keio University School of Medicine, Shinjuku, Tokyo, Japan; 5 Division of Electrical, Electronic and Information Engineering, Graduate School of Sciences and Technology for Innovation, Yamaguchi University, Ube, Yamaguchi, Japan; 6 Department of Computer Science and Electronic Engineering, National Institute of Technology, Tokuyama College, Shunan, Yamaguchi, Japan; 7 Department of Oncology Center, Yamaguchi University Hospital, Ube, Yamaguchi, Japan; 8 Department of Digestive Surgery, Kawasaki Medical University, Kurashiki, Okayama, Japan

**Keywords:** Colorectal cancer, Discrete Bayes decision rule, Prognosis, CD4, Forkhead box P3

## Abstract

**Background:**

We recently reported the relapse-free survival (RFS) significance of the combination of CD4^+^ and forkhead box P3^+^ (FOXP3) T-cell densities identified by immunohistochemistry in patients with stage I, II, and III colorectal cancer (CRC) who underwent curative resections. This study was designed to determine the optimal combination of markers that predict recurrence in patients with T factors of T3/T4a stage II CRC by applying a novel Bayes decision rule.

**Methods:**

Using 137 cancer tissue specimens from T3/T4a stage II patients, 12 clinicopathologic and immune factors were analysed as predictive candidates for recurrence.

**Results:**

Our study showed that the combination of low CD4^+^ and low FOXP3^+^ T-cell densities resulted in extremely poor RFS.

**Conclusions:**

Adjuvant chemotherapy may be considered for patients with a combination of low CD4^+^ and low FOXP3^+^ T-cell densities. The discovery of this new prognostic indicator is important for the appropriate management of patients undergoing curative resection for T3/T4a stage II CRC.

**Supplementary Information:**

The online version contains supplementary material available at 10.1186/s12885-022-10181-7.

## Introduction

Colorectal cancer (CRC) ranks third in incidence and second in mortality among cancer types globally, with more than 1.8 million new cases and over 800,000 deaths from the disease each year [1]. The most commonly used guidelines for estimating the outcomes of CRC patients that have been used for over 80 years are those of the Union for International Cancer Control tumour, node, and metastasis (TNM) classification [2, 3]. However, it is now recognised that the clinical outcome varies significantly among patients within the same stage [4–6].

Postoperative treatment with fluorouracil (FU)-based adjuvant chemotherapy has been widely used for CRC patients. Specifically, fluoropyrimidines alone or in combination with oxaliplatin regimens are the standard of adjuvant chemotherapy after resection for patients with stage III CRC [7]. Adding oxaliplatin to capecitabine has also improved disease-free survival [8] and overall survival (OS) [9]. However, the advantage of administering adjuvant chemotherapy for patients with N0 stage II CRC, which accounts for one-third of curative resections [10], remains under debate [11, 12]. Currently, high-risk stage II patients, for example, with primary tumour lesions, tumour presentation with perforation, and poorly differentiated histology [13–15], are treated with adjuvant chemotherapy, but evidence from randomised clinical trials has not shown that this confers a survival benefit [16]. Thus, novel indications of whether or not to administer adjuvant chemotherapy are required for patients with stage II CRC. Especially in T4 N0 stage II CRC, it is known that T4b is associated with an increased recurrence rate and reduced survival [17, 18]. Thus, it is important to identify prognostic factors that can allow making a decision to apply adjuvant therapy in T3 and T4a stage II CRC.

We reported a scoring system called an immunoscore that calculates the densities of tumour-infiltrating lymphocytes (TILs) within the tumour. Its invasive margins showed its usefulness for predicting the clinical outcomes of CRC patients by summarising the densities of CD3^+^ and CD8^+^ T-cell effectors [19]. Instead of applying the immunoscore, we recently reported for the first time that the combination of low CD4^+^ and low forkhead box P3^+^ (FOXP3^+^) T-cell densities exhibited extremely poor prognoses in terms of relapse-free survival (RFS) and cancer-specific survival (CSS) in 342 patients with stage I, II, and III CRC that had undergone curative resections [20].

The discrete Bayes decision rule was proposed and applied to predict the early recurrence of liver cancer [21] and lymph node metastasis in early gastric cancer [22] with high accuracy for personalised medicine. This method converts quantitative data into qualitative data by finding a certain cut-off value. The patient is then represented as a discretised data vector. To solve the two-class problem that predicts recurrence, the classifier is designed based on the discrete Bayes decision rule that can apply discrete data, and the resulting classifier discriminates between the presence and absence of recurrence with high accuracy. To handle high-dimensional data vectors, the optimal combination of markers is also selected by feature selection [21].

This study determined the optimal combination of markers that predict the recurrence of patients with T factors of T3/T4a stage II CRC by applying the discrete Bayes decision rule. Surprisingly, we found that the combination of CD4 and FOXP3 predicts recurrence, which is currently difficult to predict with the TNM staging system.

## Methods

### Patients and tissue samples

Overall, 137 cancer tissue specimens of T factor T3/T4a stage II patients who did not receive neoadjuvant chemoradiotherapy were obtained from a consecutive series of patients who underwent curative resections for CRC at the Department of Gastroenterological, Breast and Endocrine Surgery, Yamaguchi University Graduate School of Medicine, Japan from 1993 to 2012. To determine the 5-year RFS, patients who died from other diseases or stopped follow-up within 5 years after resection were excluded.

The ethics committee of Yamaguchi University Hospital (H17-83 and H23-135) approved the ethical, legal, and social implications of the study. All the samples were obtained with informed consent from the patients.

### Immunohistochemistry (IHC) and TIL analysis

Here, we briefly describe the IHC and TIL analysis method as we previously reported [20]. IHC was performed on 4-μm formalin-fixed, paraffin-embedded sections of tumour specimens. The specimens were subjected to haematoxylin and eosin staining and IHC for CD3, CD4, CD8, and FOXP3. IHC staining was performed automatically using the Ventana Discovery XT staining system (Ventana, Tucson, AZ, USA).

The following antibodies were used: anti-CD3 (rabbit monoclonal, 518,110,079, Ventana), anti-CD4 (mouse monoclonal, 518,108,816, Ventana), anti-CD8 (mouse monoclonal, 1:50, IR623, Dako, Foster City, CA, USA), and anti-FOXP3 (mouse monoclonal, 1:100, ab20034, Abcam, Cambridge, MA, USA). Furthermore, an anti-mouse Immunoglobulin G1 antibody (1:100, ab9135, Abcam) was used as an isotype control. The slides were scanned using a high-resolution digital slide scanner (NanoZoomer-XR C12000, Hamamatsu Photonics, Hamamatsu, Japan). All tumour lesions were scored automatically using a computerised image analysis system (Tissue Studio, Definiens, Munich, Germany). Measurements were recorded as the mean number of positive cells per tumour tissue unit in square millimetres [20] and the number of positive cells among each 1-mm^2^ tissue unit. Lymphoid organs, necrotic tissue, or thick fibrous tissue may be included in the collected CRC tissue. Only the main cancer lesions that did not contain peritumoural lymphocyte infiltration and extratumoural lymphoid structures were selected for this study (Fig. S1). The median and the maximum numbers of examined sections were one and four lesions, respectively.

### Statistical data analysis

#### Discrete Bayes decision rule and selection of the optimal combination of markers

The discrete Bayes decision rule [21] performs statistical decision-making based on the posterior probabilities of two classes using categorical data. This classifier rule distinguishes patients into a class with a maximum posterior probability calculated by Bayes’ theorem.

$$P\left({\omega }_{i}|{\varvec{X}}\right)=\frac{P\left({\omega }_{i}\right)P\left({\varvec{X}}|{\omega }_{i}\right)}{P\left({\omega }_{1}\right)P\left({\varvec{X}}|{\omega }_{1}\right)+P\left({\omega }_{2}\right)P\left({\varvec{X}}|{\omega }_{2}\right)},$$

where ${\varvec{X}}$ is the pattern vector that represents the patient with markers, $P\left({\omega }_{i}\right)$ is the prior probability for the recurrence or non-recurrence class, and $P\left({\varvec{X}}|{\omega }_{i}\right)$ is the class-conditional probability of class ${\omega }_{i}$. In this study, we assumed that the events in which the categorised data belong to any of the discretised divisions are mutually independent, and the prior probability is equal to 0.5 to deal with the recurrence and non-recurrence classes equally. Hence, the posterior probability is simplified as.

$$P\left({\omega }_{i}|{\varvec{X}}\right)=\frac{P\left({\varvec{X}}|{\omega }_{i}\right)}{P\left({\varvec{X}}|{\omega }_{1}\right)+P\left({\varvec{X}}|{\omega }_{2}\right)}.$$

Using the discrete Bayes decision rule, a pattern ${\varvec{X}}$ is classified into a class ${\omega }_{i}$ where the posterior probability $P\left({\omega }_{i}|{\varvec{X}}\right)$ is maximum.

The following 12 clinicopathologic and immune factors were used as predictive candidates for recurrence: age, sex, T factor, histologic grade, vascular/lymphatic invasion, tumour location, perforation, adjuvant therapy, and levels of CD3, CD4, CD8, and FOXP3. The combination of markers out of these 12 candidate markers for which *F*-measure, the harmonic mean of sensitivity and predictive value positive, that is, $$\frac{2}{{sensitivity}^{-1}+{predicitive value positive}^{-1}}$$, was maximum was selected and examined.

**Fig. 1:**
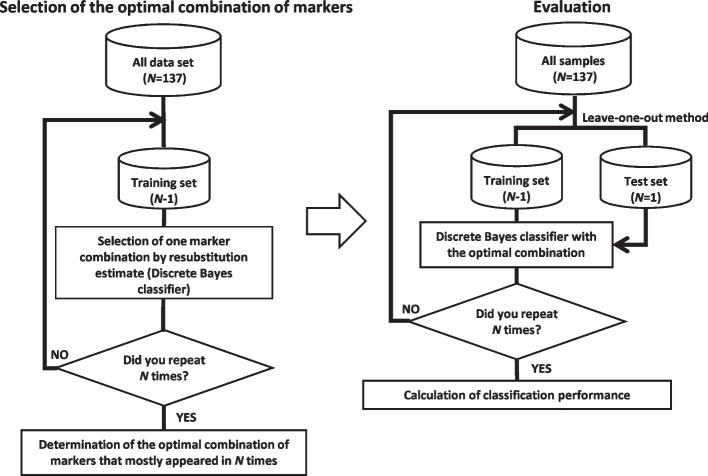
Selection of optimal combination of markers. The LOO method was applied twice to find the optimal combination of markers and to evaluate marker combinations

In pattern recognition fields, it is well known that markers cannot be selected based on individual effectiveness. Therefore, a combination of markers should be considered. To perform robust marker selection, we adopted the leave-one-out (LOO) method [23], considering the virtual variability of samples [21]. The LOO method was applied twice to find the optimal combination of markers and evaluate the marker combinations obtained by the algorithm (Fig. 1).

A total of 137 samples were collected. To explore the marker combinations, initially, one combination of two markers out of 12 candidate markers was selected, and then one patient out of the 137 was selected as a test sample based on the LOO method, and the remaining 136 patients were assigned as training samples. The discrete Bayes classifier was designed using these 136 training samples, and this classifier distinguished the same training samples. Note that one test sample was not used for the marker selection. The resubstitution method evaluated each of the combinations of two markers based on the discrete Bayes classifier [23]. From the evaluation results, one combination which satisfied the condition that maximal sensitivity, subject to specificity $\ge 50\%$ or maximal *F*-measure, was selected. This process was repeated until each sample was used only once as a test sample. We determined the optimal combination of markers that mostly appeared in 137 attempts.

According to the above procedure, marker selection was conducted again when the number of markers was three. The discrete Bayes decision rule and LOO algorithm were computed using the R statistical programming language (version 3.6.1) [24].

##### Survival analysis

RFS was defined as the interval from the date of curative resection of CRC to the date of cancer recurrence diagnosis. Survival curves were estimated using the Kaplan–Meier (KM) method and tested using the log-rank test. Statistical tests were performed using R (version 3.6.1) [24]. A value of *p* < 0.05 was considered indicative of statistical significance.

## Results

### Patient characteristics

Table 1 shows the characteristics of the 137 patients with T3/T4a stage II CRC. Among these patients, a 5-year recurrence was observed in 34 (24.8%). The median age was 71.0 years (interquartile range, 62.0–78.0 years). There were 73 (53.3%) men and 64 (46.7%) women. One hundred and sixteen (84.7%) and 21 patients (15.3%) had T3 and T4a stage II CRC, respectively. Regarding histologic grades, 25 tumours (18.2%) were well-differentiated, 104 (75.9%) were moderately differentiated, 3 (2.2%) were poorly differentiated, 2 (1.5%) were mucinous, and 3 (2.2%) were undifferentiated. Vascular/lymphatic invasion was observed in 70 patients (51.1%). Eighty-one (59.1%) and 56 patients (40.9%) had tumours on the left and right sides, respectively. Two patients (1.5%) had perforation, and 87 (63.5%) received adjuvant therapy with tegafur/uracil (UFT), UFT/leucovorin calcium (LV), 5-FU, 5-FU/LV, doxifluridine, or tegafur/gimeracil/oteracil (TS-1).

**Table 1:** Patient characteristics (Stage II, T factor: T3/T4a)

Variables	*n* = 137
Age^♦^	71.0 [62.0, 78.0]
Sex: male/female	73/64
T factor: T3/T4a	116/21
Histologic grade	
well	25
moderately	104
poorly	3
mucinous	2
undifferentiated	3
Vascular/lymphatic invasion: present/absent	70/67
Location: left side/right side	81/56
Perforation: present/absent	2/135
Adjuvant therapy: Yes/No	87/50
5-year reccurence: Yes/No	34/103

^♦^median [interquartile range]

### Optimal marker combinations

Table 2 shows the relationship between clinicopathologic/immunological characteristics and 5-year recurrence rates. Using 137 available samples, the combination of markers included CD4 and FOXP3 for two markers and CD4, FOXP3, and histologic grade for three markers (Table 3). These markers were determined to be the optimal combinations of markers. According to the LOO method with 137 samples, the discrete Bayes classifier was designed with these combinations, and then the classification performance was evaluated (Fig. 1). The combination of two markers on the discrete Bayes classifier produced 68% and 69% sensitivity and specificity, respectively (an area under the curve [AUC] of 0.68: Fig. S2-A). More precisely, Table 3 shows the diagnostic potential of recurrence using the discrete Bayes classifier. For instance, assuming that a patient was classified as having low CD4^+^ and low FOXP3^+^ TIL densities, the classifier-distinguished recurrence resulted in 100% sensitivity (23/23) and 0% specificity (0/32). We could thus differentiate recurrence with a sensitivity of 68%, a specificity of 69%, an accuracy of 69%, a predictive value of 42%, and *an F*-measure of 0.52. In contrast, the combination of three markers on the discrete Bayes classifier resulted in a sensitivity of 71%, a specificity of 67% (an AUC of 0.69: Fig. S2-B), an accuracy of 68%, a positive predictive value of 41%, and *an F*-measure of 0.52.

**Table 2:** The association of clinicopathological and immunological characteristics with 5-year recurrence

Variables	*n* = 137	
	5-year recurrence: Yes (*n* = 34)	5-year recurrence: No (*n* = 103)
Age^♦^	73.0 [63.5, 78.8]	70.0 [61.5, 77.0]
Sex: male/female	18/16	55/48
T factor: T3/T4a	28/6	88/15
Histologic grade		
well	5	20
moderately	26	78
poorly	1	2
mucinous	0	2
undifferentiated	2	1
Vascular/lymphatic invasion: present/absent	21/13	49/54
Location: left side/right side	19/15	62/41
Perforation: present/absent	0/34	2/101
Adjuvant therapy: Yes/No	24/10	63/40
CD3 density: High/Low	13/21	47/56
CD4 density: High/Low	8/26	57/46
CD8 density: High/Low	13/21	54/49
FOXP3 density: High/Low	6/28	43/60
CD4 & FOXP3 densities: Low & Low/Other combinations	23/11	32/71

^♦^median [interquartile range]; high values of CD3, CD8, CD4 nad FOXP3 are ≥ 339.1, ≥ 72.2, ≥ 64.6 and ≥ 89.1 cells/mm^2^, respectively

**Table 3:** Classification results (Stage II, T factor: T3/T4a)

**Optimal combination of markers**					
**CD4**	**FOXP3**		**Decision**	**Sensitivity**	**Specificity**
High	High		Non-recurrence	0 (0/3)	1 (29/29)
High	Low		Non-recurrence	0 (0/5)	1 (28/28)
Low	High		Non-recurrence	0 (0/3)	1 (14/14)
Low	Low		Recurrence	1 (23/23)	0 (0/32)
			**Total**	0.68 (23/34)	0.69 (71/103)
**CD4**	**FOXP3**	**Histologic grade**			
High	High	Well/Moderate	Non-recurrence	0 (0/3)	1 (28/28)
High	Highb	Other	Recurrence		0 (0/1)
High	Low	Well/Moderate	Non-recurrence	0 (0/4)	1 (27/27)
High	Low	Other	Recurrence	1 (1/1)	0 (0/1)
Low	High	Well/Moderate	Non-recurrence	0 (0/3)	1 (14/14)
Low	High	Other			
Low	Low	Well/Moderate	Recurrence	1 (21/21)	0 (0/29)
Low	Low	Other	Recurrence	1 (2/2)	0 (0/3)
			**Total**	0.71 (24/34)	0.67 (69/103)

The relationship between the clinicopathological and immunological characteristics and the presence of adjuvant therapy is shown in Table S1. The results of 50 independent discrimination tests in the absence group of adjuvant therapy samples also selected the combination of CD4 and FOXP3 for the combination of two markers and CD4, FOXP3, and histologic grade for the combination of three markers. The discrete Bayes classifier resulted in 80% sensitivity (8/10) and 70% specificity (28/40) for the two markers and 90% sensitivity (9/10) and 68% specificity (27/40) for the three markers (Table S2).

### Survival analysis

**Fig. 2:**
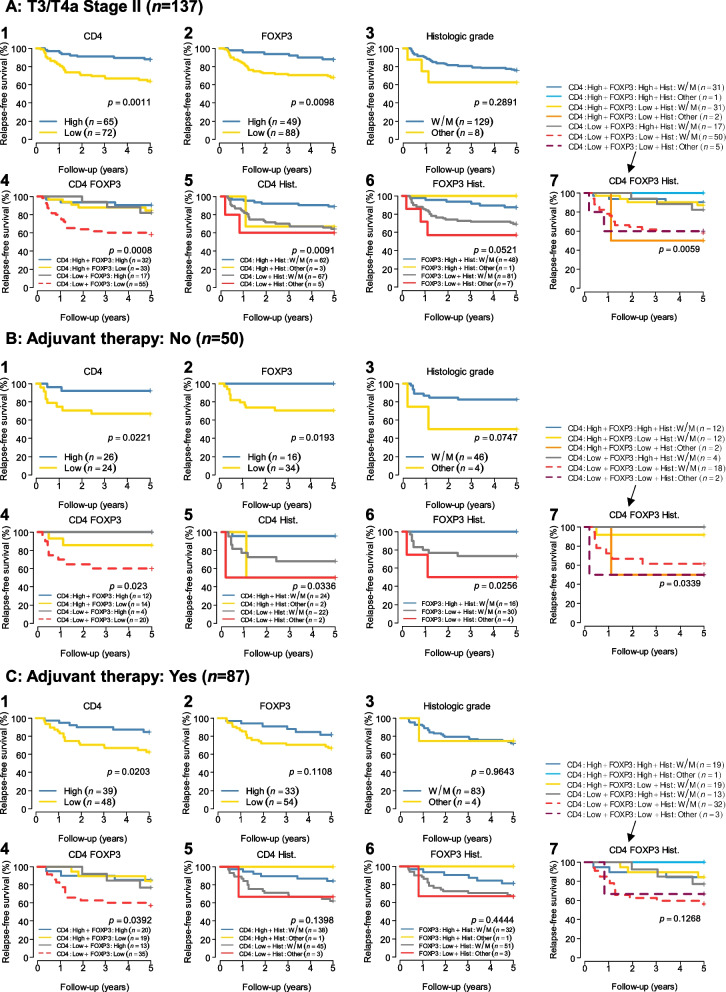
Survival analysis after surgery based on the optimal maker subsets of TILs. **A** RFS based on the total number of patients. **B** RFS based on the absence of adjuvant therapy. **C** RFS based on the presence of adjuvant therapy. The cut-off values to distinguish high and low cell densities were 339.1, 72.2, 64.6, and 89.1 cells/mm^2^ for CD3, CD8, CD4, and FOXP3, respectively. W/M, well/moderately differentiated; Other, poorly differentiated/mucinous/undifferentiated; Hist., histologic grade; *p*-value, log-rank test

In total, there were 34 CRC-specific recurrences. The KM survival curves are shown in Fig. 2A, according to the optimal marker combination results. The log-rank tests showed that high CD4^+^ ($p=0.0011;$ Fig. 2A-1) and FOXP3^+^ cell densities ($p=0.0098;$ Fig. 2A-2) were associated with improved RFS. Notably, a combination of CD4^+^ and FOXP3^+^ cell densities most precisely predicted the prognosis ($p=0.0008;$ Fig. 2A-4).

Patients with a combination of low CD4^+^ and low FOXP3^+^ TIL density were associated with remarkably poor 5-year RFS (58.18% [95% confidence interval: 46.50–72.79]) compared to patients with a combination of high CD4^+^ and FOXP3^+^ TIL densities (90.62% [81.07–100]), high CD4^+^ and low FOXP3^+^ TIL densities (84.85% [73.46–98.01]) or low CD4^+^ and high FOXP3^+^ TIL densities (82.35% [66.09–100]).

Similarly, for the absence group of adjuvant therapy (Fig. 2B), the log-rank tests showed that a high CD4^+^ cell density ($p=0.0221;$ Fig. 2B-1) and FOXP3^+^ cell density ($p=0.0193;$ Fig. 2B-2) were associated with improved RFS, and patients with a combination of low CD4^+^ and FOXP3^+^ TIL densities were associated with poor RFS ($p=0.023;$ 5-year RFS: 60.0% [42.0–85.80], Fig. 2B-4). Figure 2C-4 shows that the combination of low CD4^+^ TIL density and low FOXP3^+^ TIL density was significantly associated with poor RFS in the adjuvant therapy group ($p=0.0392$, 5-year RFS: 57.14% [42.89–76.13]). In addition, Fig. S3A-4 shows that the combination of low CD4^+^ TIL density and low FOXP3^+^ TIL density was significantly associated with poor RFS in the tumours on the left side.

## Discussion

This study applying the novel Bayes decision rule showed the RFS significance of the combination of CD4^+^ and FOXP3^+^ T-cell densities identified by IHC in T3/T4a stage II CRC patients. Adjuvant chemotherapy may be considered for patients with a combination of low CD4^+^ TIL density and low FOXP3^+^ TIL density, and fluoropyrimidines in combination with oxaliplatin regimens are recommended for such candidates.

We distinguished recurrence in T3/T4a stage II CRC by applying a novel discrete Bayes classifier. This classifier is unique and can deal with numerical and non-numerical data based on the Bayes decision theory using the posterior probability [21]. To find the optimal combination of markers and evaluate distinguishability, we applied the LOO method. Using this estimation method, we eliminated the arbitrariness in recurrence classification and obtained an objective probability distribution. Our results showed that CD4 and FOXP3 for two combination markers and CD4, FOXP3, and histologic grade for three combination markers could predict recurrence most significantly. If a clinician determines a patient as experiencing recurrence, they can differentiate the recurrence with a sensitivity of 68 and 71%, a specificity of 69 and 67%, and a diagnostic accuracy rate of 69 and 68% for the two and three combination markers, respectively (Table 3).

As shown in Table S2, the classification performance for the absence group of adjuvant therapy using two and three optimal combinations of markers against test samples showed sensitivities of 80 and 90% and a specificity of 70 and 68%, respectively. The discrete Bayes classifier resulted in high sensitivity, an important indicator for predicting CRC recurrence. Since we attempted not to miss recurrence, we adopted an evaluation standard in which sensitivity is maximised by maintaining the specificity at a certain level.

Consistent with our previous report [20], the present study confirmed the usefulness of CD4^+^ T-cell density itself as a prognostic factor (Fig. 2A-1, B-1, and C-1), and the combination of low CD4^+^ cell infiltration and low FOXP3^+^ cell infiltration was a prognostic factor for low RFS in T3/T4a stage II CRC patients (Fig. 2A-4, B-4 and C-4).

We first showed that a high CD4^+^ T-cell density was associated with a longer RFS. To our knowledge, this is the first report to suggest the usefulness of intratumoural CD4^+^ T-cell infiltration as a positive RFS factor in T3/T4a stage II CRC. It has been reported that in lung, renal, prostate, and breast cancers, CD4^+^ T-cell density is a negative prognostic factor [25–28]. Hence, a higher recurrence risk was expected in the case of higher CD4^+^ expression. The reason for this discrepancy between CRC and other cancers remains ambiguous. However, possible differences in the function of CD4^+^ T cells within the tumour microenvironment, such as immune response activation or immunosuppression depending on the cancer type, can be one of the reasons, as we reported previously [20].

Poor clinical outcomes in many cancers are associated with the invasion of abundant FOXP3^+^ cells into the tumour tissue [29]; therefore, higher recurrence risk is also expected in the case of higher FOXP3^+^ expression in CRC. However, contradictory results have been reported in CRC; cases with high FOXP3^+^ T-cell infiltration showed better prognosis in some studies [30–32]. According to our results, a high FOXP3^+^ cell density was significantly associated with improved prognosis (Fig. 2A-2 and B-2).

Finally, we found that the combination of CD4^+^ and FOXP3^+^ cell densities was a precise prognostic marker (Fig. 2A-4, B-4, and C-4). CD4^+^ T cells that express the FOXP3 transcription factor function as Treg cells that suppress effective immune responses against cancer cells [33, 34]. FOXP3^+^/CD4^+^ T cells are both functionally and phenotypically heterogeneous; FOXP3^+^/CD4^+^ T cells can be fractionated based on their expression levels of FOXP3 and CD45RA into FOXP3^low^/CD45RA^+^ naive Treg cells, FOXP3^high^/CD45RA^−^ effector Treg cells, and FOXP3^low^/CD45RA^−^ non-suppressive T cells that can secrete proinflammatory cytokines [34–36]. Our results indicate that the infiltration of only one type of immune cell, such as CD4^+^ or FOXP3^+^ cells, might be sufficient for a suitable tumour microenvironment to prevent recurrence. Although further studies are required to clarify the mechanism underlying these results, including the effect of tumour location (Fig. S3A-4 and B-4), our findings offer new ideas and insights on tumour immunity.

In the QUASAR trial, the chemotherapy group (5-FU + LV $\pm$ levamisole) had better RFS and OS compared to the surgery-only group in stage I, II, and III CRC but no significant advantage in stage II CRC itself [37]. Furthermore, the IMPACT B2 trial [38] and its meta-analysis [39, 40] and SEER database review [41] reported no significant advantage in the chemotherapy group (5-FU + LV) in RFS and OS for patients with T3N0 CRC. In addition, the SACURA trial showed no significant advantage for applying adjuvant chemotherapy (UFT) to stage II colon cancer in terms of RFS [42]. Hence, it is difficult to apply adjuvant therapy to every stage II CRC patient without considering recurrence risks.

Based on the American Society of Clinical Oncology (ASCO) and the European Society for Medical Oncology (ESMO) guidelines, it is recommended to apply adjuvant chemotherapy in high-risk stage II CRC patients. Poor prognostic features such as fewer than 12 retrieved lymph nodes, T4 lesions (defined as adherence to or invasion of local organs), tumour presentation with perforation and poorly differentiated adenocarcinoma/signet ring cell carcinoma/mucinous carcinoma, and T4, poorly differentiated adenocarcinoma/undifferentiated cancer, lymphovascular invasion, paranerve infiltration, intestinal obstruction/perforation, and fewer than 12 retrieved lymph nodes from the ASCO 2004 [43] and ESMO guidelines [15], respectively, are known as high-risk factors for stage II CRC. Thus, it is essential to identify prognostic factors that can influence the decision to apply adjuvant therapy in T3 and T4a stage II CRC. In this regard, our CD4 and FOXP3 combination could be a novel prognostic factor.

One of the main limitations of this study was the use of a small amount of sample data from a single institution. Nevertheless, the LOO method was used to obtain highly accurate results, even with few samples. Each sample was used as a training sample and as a test sample, but never as a training sample or a test sample at the same time, and the LOO method was excellent in terms of use efficiency and independence. Therefore, high estimated accuracy is expected. There is room for developing more accurate criteria by assessing more cases with multiple institutions to establish more precise criteria, and we aim to conduct a prospective study to verify the validity of this study’s findings.

## Conclusions

We believe that this study applying the novel Bayes decision rule is the first to report the RFS significance of the combination of CD4^+^ and FOXP3^+^ T-cell densities identified by IHC in T3/T4a stage II CRC patients. The discovery of this new prognostic indicator is essential for appropriately managing patients undergoing curative resection for high-risk stage II CRC.

## Supplementary Information

**Additional file 1:** **Fig. S1.**  IHC of CD3^+^ TILs in CRC. A) Colon tissues were divided into 1-mm^2^ tiles, with tumour tissue highlighted in red. B) Tumour regions were selected as the area under the curve (indicated by arrows), excluding peritumoural lymphocyte infiltration (open triangle) and extratumoural lymphoid structures (closed triangle). C) Representative IHC showing high CD3^+^ cell density. D) Representative IHC showing low CD3^+^ cell density. 

**Additional file 2:** **Fig. S2. **Receiver operating characteristic curves based on the optimal maker subsets of TILs. A) The combination of two markers. B) The combination of three markers. AUC, area under the curve; CI, confidence interval. **Fig. S3.** Survival analysis after surgery based on the optimal maker subsets of TILs. A) RFS based on the tumours on the left side. B) RFS based on the tumours on the right side. W/M, well/moderately differentiated; Other, poorly differentiated/mucinous/undifferentiated; Hist., histologic grade; *p*-value, log-rank test.

**Additional file 3:** **Table S1.** The association of clinicopathological and immunological characteristics with adjuvant therapy status (Stage II, T factor: T3/T4a). **Table S2.** Classification results (Stage II, T factor: T3/T4a, Adjuvant therapy: No). 

## Data Availability

Data are available upon request (to Yuki Nakagami; nakagami-yu@shimonoseki-cu.ac.jp). The study data are considered commercially proprietary and are not stored for unrestricted access. All authors have full access to the study data and take responsibility for the integrity of the data and the accuracy of the data analysis.

## Abbreviations

RFS: Relapse-free survival
FOXP3: Forkhead box P3
CRC: Colorectal cancer
TNM: Tumour, node, and metastasis
FU: Fluorouracil
OS: Overall survival
TIL: Tumour-infiltrating lymphocyte
CSS: Cancer-specific survival
IHC: Immunohistochemistry
LOO: Leave-one-out
KM: Kaplan–Meier
ASCO: The American Society of Clinical Oncology
ESMO: The European Society for Medical Oncology
