# Supplementary material for: CD4 and FOXP3 as predictive markers for the recurrence of T3/T4a stage II colorectal cancer: applying a novel discrete Bayes decision rule
Source: BMC Cancer. 2022 Oct 18;22:1071. doi: 10.1186/s12885-022-10181-7 (PMC9578193; doi:10.1186/s12885-022-10181-7)
Supplement: Supplementary file 1 — Additional file 1: Fig. S1. IHC of CD3+ TILs in CRC. A) Colon tissues were divided into 1-mm2 tiles, with tumour tissue highlighted in red. B) Tumour regions were selected as the area under the curve (indicated by arrows), excluding peritumoural lymphocyte infiltration (open triangle) and extratumoural lymphoid structures (closed triangle). C) Representative IHC showing high CD3+ cell density. D) Representative IHC showing low CD3+ cell density. [file 12885_2022_10181_MOESM1_ESM.pdf]

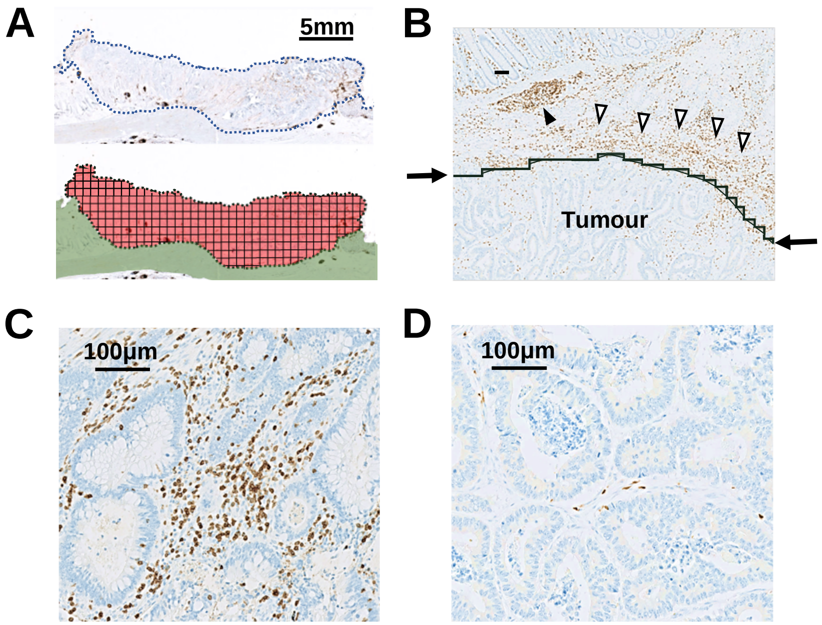

Fig. S1. IHC of CD3^{+} TILs in CRC

A) Colon tissues were divided into 1-mm^{2} tiles, with tumour tissue highlighted in red. **B)** Tumour regions were selected as the area under the curve (indicated by arrows), excluding peritumoural lymphocyte infiltration (open triangle) and extratumoural lymphoid structures (closed triangle). **C)** Representative IHC showing high CD3^{+} cell density. **D)** Representative IHC showing low CD3^{+} cell density.
