# Supplementary material for: CD4 and FOXP3 as predictive markers for the recurrence of T3/T4a stage II colorectal cancer: applying a novel discrete Bayes decision rule
Source: BMC Cancer. 2022 Oct 18;22:1071. doi: 10.1186/s12885-022-10181-7 (PMC9578193; doi:10.1186/s12885-022-10181-7)
Supplement: Supplementary file 2 — Additional file 2: Fig. S2. Receiver operating characteristic curves based on the optimal maker subsets of TILs. A) The combination of two markers. B) The combination of three markers. AUC, area under the curve; CI, confidence interval. Fig. S3. Survival analysis after surgery based on the optimal maker subsets of TILs. A) RFS based on the tumours on the left side. B) RFS based on the tumours on the right side. W/M, well/moderately differentiated; Other, poorly differentiated/mucinous/undifferentiated; Hist., histologic grade; p-value, log-rank test. [file 12885_2022_10181_MOESM2_ESM.pdf]

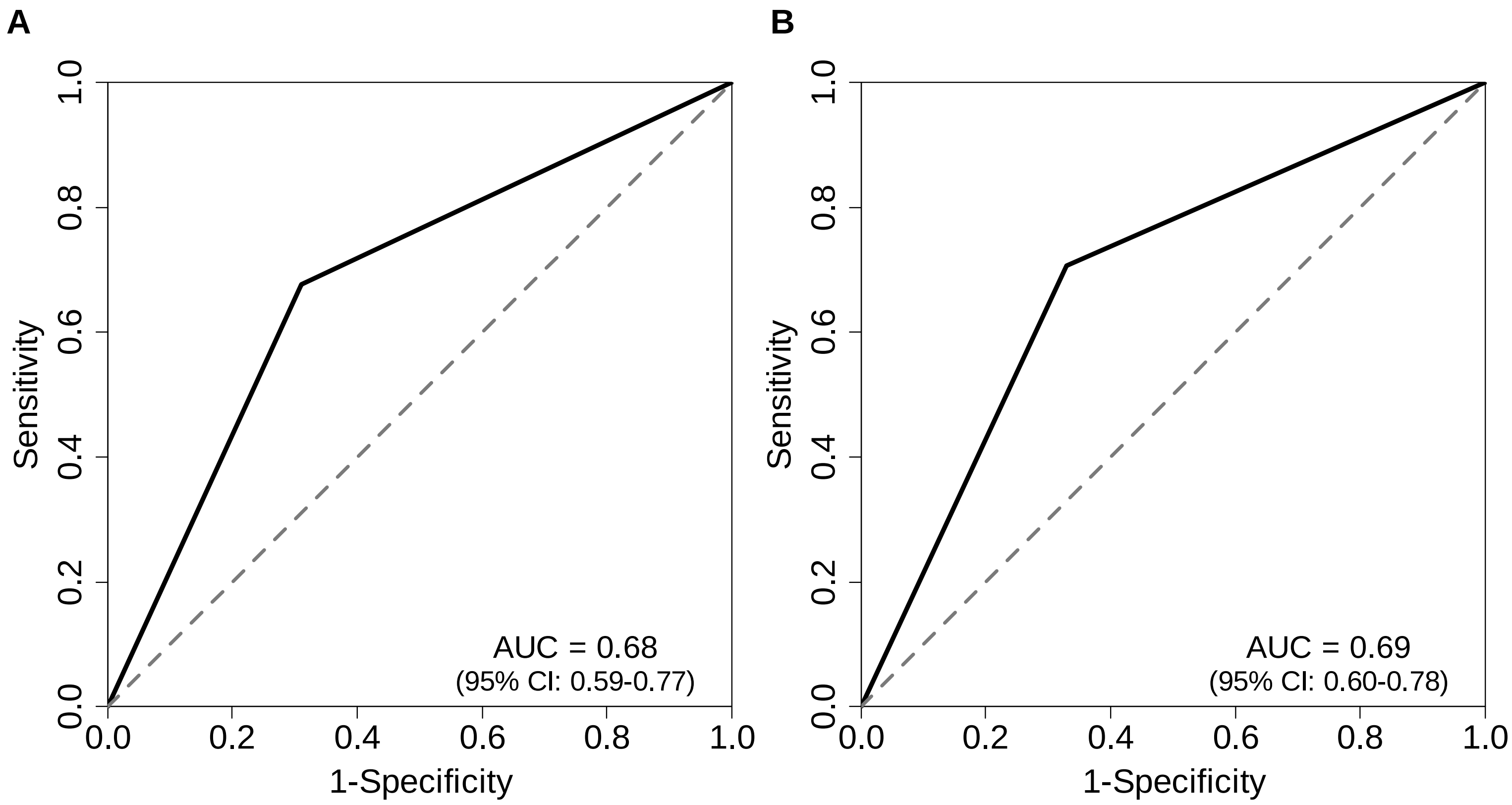

Fig. S2. Receiver operating characteristic curves based on the optimal maker subsets of TILs

A) The combination of two markers. **B)** The combination of three markers. AUC, area under the curve; CI, confidence interval.

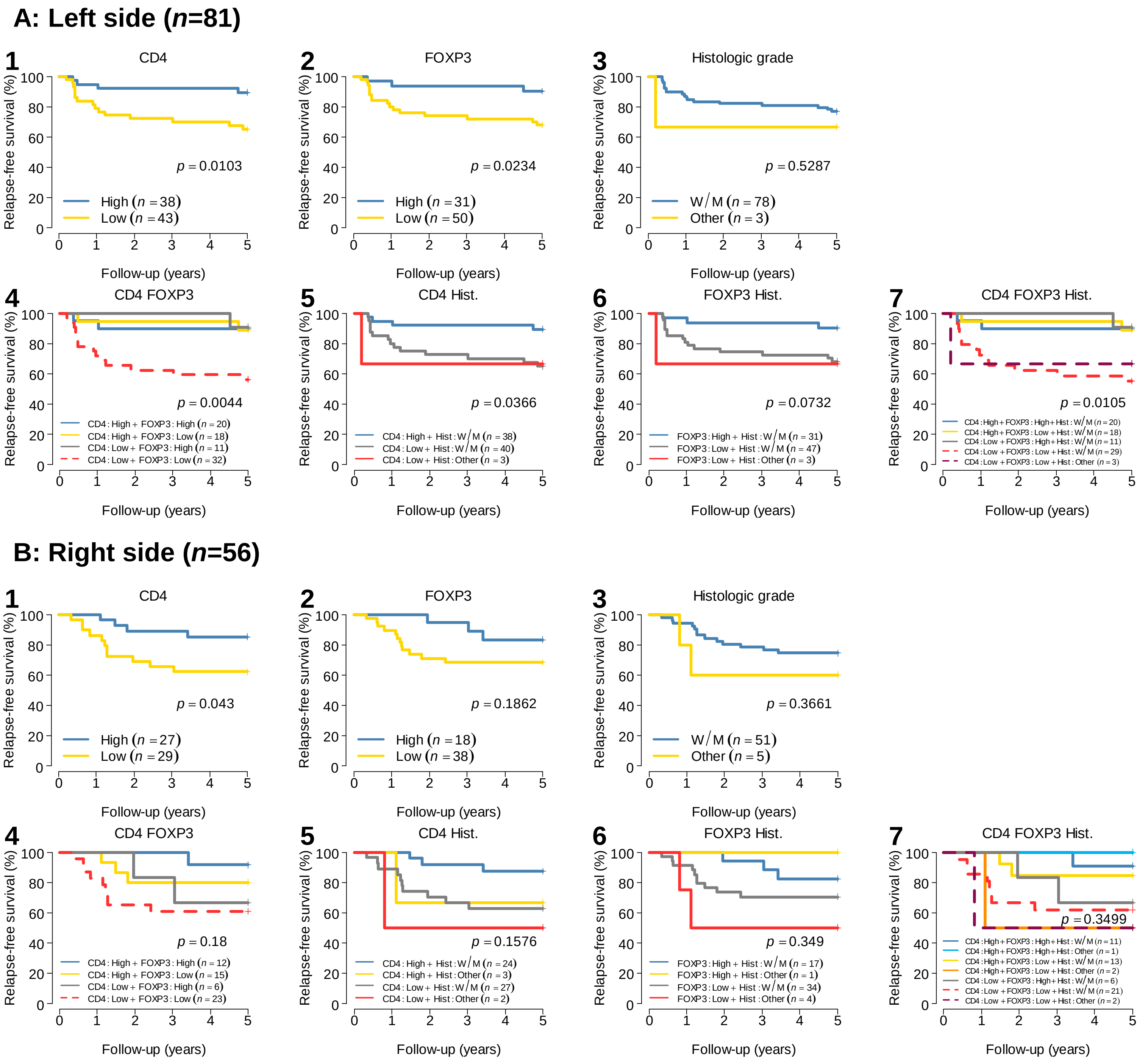

Fig. S3. Survival analysis after surgery based on the optimal maker subsets of TILs

A) RFS based on the tumours on the left side. **B)** RFS based on the tumours on the right side. W/M, well/moderately differentiated; Other, poorly differentiated/mucinous/undifferentiated; Hist., histologic grade; *p*-value, log-rank test.
