# Supplementary material for: CD4 and FOXP3 as predictive markers for the recurrence of T3/T4a stage II colorectal cancer: applying a novel discrete Bayes decision rule
Source: BMC Cancer. 2022 Oct 18;22:1071. doi: 10.1186/s12885-022-10181-7 (PMC9578193; doi:10.1186/s12885-022-10181-7)
Supplement: Supplementary file 3 — Additional file 3: Table S1. The association of clinicopathological and immunological characteristics with adjuvant therapy status (Stage II, T factor: T3/T4a). Table S2. Classification results (Stage II, T factor: T3/T4a, Adjuvant therapy: No). [file 12885_2022_10181_MOESM3_ESM.pdf]

Table S1. The association of clinicopathological and immunological characteristics with adjuvant therapy status (Stage II, T factor: T3/T4a)

Variables	Adjuvant therapy: Yes (*n* =87)		Adjuvant therapy: No (*n* =50)	
	5-year recurrence: Yes (*n* =24)	5-year recurrence: No (*n* =63)	5-year recurrence: Yes (*n* =10)	5-year recurrence: No (*n* =40)
Age†	71.0 [64.5, 78.2]	67.0 [59.0, 75.0]	76.0 [62.5, 80.2]	75.5 [63.8, 80.2]
Sex: male/female	11/13	38/25	7/3	17/23
T factor: T3/T4a	18/6	52/11	10/0	36/4
Histologic grade				
well	4	15	1	5
moderately	19	45	7	33
poorly	1	1	0	1
mucinous	0	2	0	0
undifferentiated	0	0	2	1
Vascular/lymphatic invasion: present/absent	15/9	29/34	6/4	20/20
Location: left side/right side	13/11	38/25	6/4	24/16
Perforation: present/absent	0/24	2/61	10/0	40/0
CD3 density: High/Low	11/13	29/34	2/8	18/22
CD4 density: High/Low	6/18	33/30	2/8	24/16
CD8 density: High/Low	10/14	35/28	3/7	19/21
FOXP3 density: High/Low	6/18	27/36	0/10	16/24
CD4 & FOXP3 densities: Low & Low/Other combinations	15/9	20/43	8/2	12/28

†, median [interquartile range]; high values of CD3, CD8, CD4 and FOXP3 are ≥339.1, ≥72.2, ≥64.6 and ≥89.1 cells/mm2, respectively.

Table S2. Classification results (Stage: II, T factor: T3/T4a, Adjuvant therapy: No)

Optimal combination of markers					
CD4	FOXP3	―	Decision	Sensitivity	Specificity
High	High	―	Non-recurrence	―	1 (12/12)
High	Low	―	Non-recurrence	0 (0/2)	1 (12/12)
Low	High	―	Non-recurrence	―	1 (4/4)
Low	Low	―	Recurrence	1 (8/8)	0 (0/12)
			Total	0.80 (8/10)	0.70 (28/40)
CD4	FOXP3	Histologic grade			
High	High	Well/Moderate	Non-recurrence	―	1 (12/12)
High	High	Other	―	―	―
High	Low	Well/Moderate	Non-recurrence	0 (0/1)	1 (11/11)
High	Low	Other	Recurrence	1 (1/1)	0 (0/1)
Low	High	Well/Moderate	Non-recurrence	―	1 (4/4)
Low	High	Other	―	―	―
Low	Low	Well/Moderate	Recurrence	1 (7/7)	0 (0/11)
Low	Low	Other	Recurrence	1 (1/1)	0 (0/1)
			Total	0.90 (9/10)	0.68 (27/40)
